# Protective Effect of Pretreatment with Acenocoumarol in Cerulein-Induced Acute Pancreatitis

**DOI:** 10.3390/ijms17101709

**Published:** 2016-10-12

**Authors:** Zygmunt Warzecha, Paweł Sendur, Piotr Ceranowicz, Marcin Dembiński, Jakub Cieszkowski, Beata Kuśnierz-Cabala, Rafał Olszanecki, Romana Tomaszewska, Tadeusz Ambroży, Artur Dembiński

**Affiliations:** 1 Department of Physiology, Faculty of Medicine, Jagiellonian University Medical College, 16 Grzegórzecka St., 31-531 Cracow, Poland; p.send@interia.pl (P.S.); mpcerano@cyf-kr.edu.pl (P.C.); jakub.cieszkowski@uj.edu.pl (J.C.); mpdembin@cyf-kr.edu.pl (A.D.); 2 Department of Anesthesiology and Intensive Therapy, Faculty of Medicine, Jagiellonian University Medical College, 31-501 Cracow, Poland; 3 The Second Department of General Surgery, Faculty of Medicine, Jagiellonian University Medical College, 31-501 Cracow, Poland; mpmdembi@cyf-kr.edu.pl; 4 Department of Clinical Biochemistry, Faculty of Medicine, Jagiellonian University Medical College, 31-501 Cracow, Poland; mbkusnie@cyf-kr.edu.pl; 5 Department of Pharmacology, Faculty of Medicine, Jagiellonian University Medical College, 31-531 Cracow, Poland; rafal.olszanecki@uj.edu.pl; 6 Department of Pathology, Faculty of Medicine, Jagiellonian University Medical College, 31-531 Cracow, Poland; romana.tomaszewska@uj.edu.pl; 7 Faculty of Physical Education and Sport, University of Physical Education, 31-571 Cracow, Poland; tadek@ambrozy.pl

**Keywords:** acute pancreatitis, coagulation, inflammation, interleukin-1β, lipase

## Abstract

Coagulation is recognized as a key player in inflammatory and autoimmune diseases. The aim of the current research was to examine the effect of pretreatment with acenocoumarol on the development of acute pancreatitis (AP) evoked by cerulein. Methods: AP was induced in rats by cerulein administered intraperitoneally. Acenocoumarol (50, 100 or 150 µg/kg/dose/day) or saline were given once daily for seven days before AP induction. Results: In rats with AP, pretreatment with acenocoumarol administered at the dose of 50 or 100 µg/kg/dose/day improved pancreatic histology, reducing the degree of edema and inflammatory infiltration, and vacuolization of acinar cells. Moreover, pretreatment with acenocoumarol given at the dose of 50 or 100 µg/kg/dose/day reduced the AP-evoked increase in pancreatic weight, serum activity of amylase and lipase, and serum concentration of pro-inflammatory interleukin-1β, as well as ameliorated pancreatic DNA synthesis and pancreatic blood flow. In contrast, acenocoumarol given at the dose of 150 μg/kg/dose did not exhibit any protective effect against cerulein-induced pancreatitis. Conclusion: Low doses of acenocoumarol, given before induction of AP by cerulein, inhibit the development of that inflammation.

## 1. Introduction

There is close bidirectional relationship between coagulation and inflammation [1,2]. Inflammation can be both a cause and a result of induction of coagulation [1,2,3,4]. Similarly, coagulation results not only in thrombosis but also in activation of inflammatory process [1,2,5].

Acute pancreatitis (AP) is associated with coagulative disorders [6,7,8,9]. The stage of these disorders depends on the severity of AP. Mild AP is associated with scattered intravascular thrombosis, whereas severe AP may lead to the development of disseminated intravascular coagulation [6,7,8,9]. Measurement of disseminated intravascular coagulation parameters is useful in the assessment of AP severity and prediction for poor outcome of this disease at admission [7,8].

There are animal and clinical studies showing protective and therapeutic effect of a well-known anticoagulant, heparin, in AP. Animals studies have indicated that pretreatment with heparin inhibits the development of AP evoked by bile [9], taurocholate [10], cerulein [11] and pancreatic ischemia followed by reperfusion [12]. Administration of heparin after induction of AP has been shown to accelerate the recovery in acute pancreatitis evoked by cerulein [13] and pancreatic ischemia followed by reperfusion [12].

Clinical reports suggests that pretreatment with heparin reduces frequency of post-ERCP (endoscopic retrograde cholangiopancreatography) pancreatitis [14,15]. Moreover, treatment with heparin given together with insulin is recommended as a gold standard in the management of hyperlipidemia-induced AP [16,17,18]. Heparin has also been found to be effective in the prevention of encephalopathy in the course of severe AP [19].

Mechanism of anticoagulant activity of heparin requires the presence of antithrombin III. Antithrombin III is a protease inhibitor and complex heparin–antithrombin III inactivates thrombin and active forms of clotting factors, IX, X, XI and XII, as well as inhibits the formation of thrombin and active forms of clotting factors [20,21].

Observations concerning anti-inflammatory effects of heparin in AP led to the question whether other anticoagulants such as coumarins also exhibit anti-inflammatory activity in this disease. Coumarins are vitamin K antagonists. Reduced vitamin K is a necessary cofactor for the hepatic γ-glutamyl carboxylase activity. That enzyme adds a carboxyl group to glutamic acid residues in immature clotting factors II, VII, IX, and X as well as proteins S, C and Z. During activity of γ-glutamyl carboxylase, vitamin K is oxidized. Coumarins inhibit the vitamin K epoxide reductase, an enzyme that reduces vitamin K back to its active form. Coumarins reduce plasma levels of vitamin K-dependent clotting factors causing a reduction in blood coagulability [21,22].

Our recent study has shown that inhibition of coagulation by pretreatment with low doses of acenocoumarol, a drug from the group of coumarins, exhibits protective effect in the pancreas leading to reduction of the severity of ischemia/reperfusion-induced AP [20]. On the other hand, there are no data on whether protective effect of acenocoumarol in the pancreas depends on the cause of AP or exhibits universal nature and occurs regardless of the primary agent causing this inflammation. The effect of pretreatment with acenocoumarol on the development of AP evoked by a primary non-vascular mechanism is unknown. Therefore, the aim of our present study was to investigate whether the pretreatment with low doses of acenocoumarol affect the development of cerulein-induced AP.

## 2. Results

**Figure 1:**
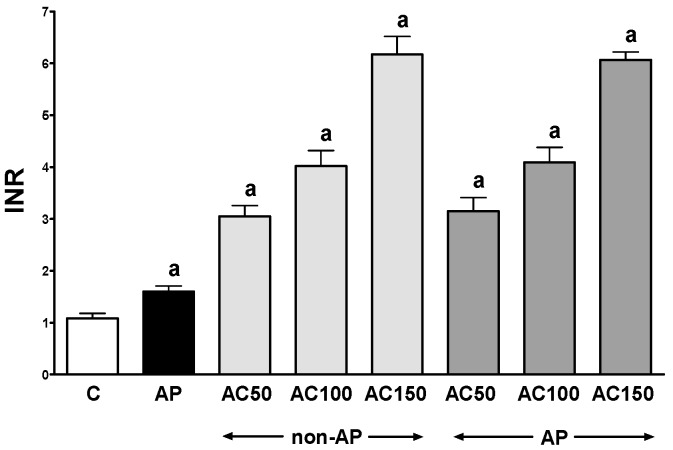
Impact of pretreatment with acenocoumarol on the prothrombin time measured as international normalized ratio (INR) in rats with or without cerulein-induced pancreatitis. Key: C = control; AP = cerulein-induced acute pancreatitis; non-AP = groups without induction of acute pancreatitis; AC = acenocoumarol; 50 = 50 μg/kg/day; 100 = 100 μg/kg/day; 150 = 150 μg/kg/day. Mean ± SEM. *n* = 10 in each group of rats. ^a^ *p* < 0.05 compared to control.

**Figure 2:**
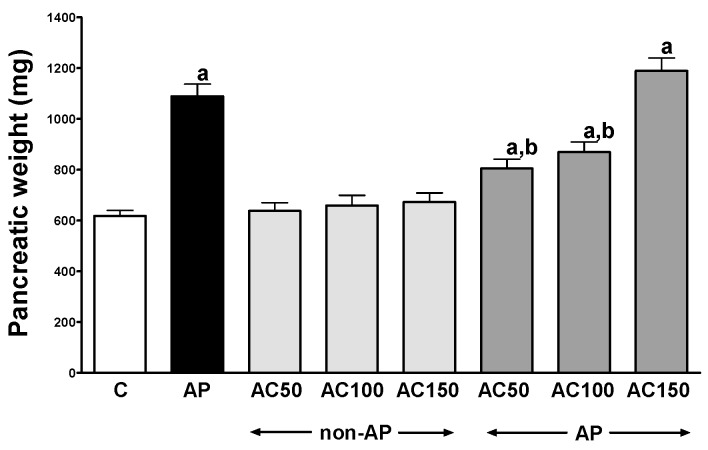
Impact of pretreatment with acenocoumarol on the weight of the pancreas in rats with or without cerulein-induced pancreatitis. Key: C = control; AP = cerulein-induced acute pancreatitis; non-AP = groups without induction of acute pancreatitis; AC = acenocoumarol; 50 = 50 μg/kg/day; 100 = 100 μg/kg/day; 150 = 150 μg/kg/day. Mean ± SEM. *n* = 10 in each group of rats. ^a^ *p* < 0.05 compared to control; ^b^ *p* < 0.05 compared to AP alone.

**Figure 3:**
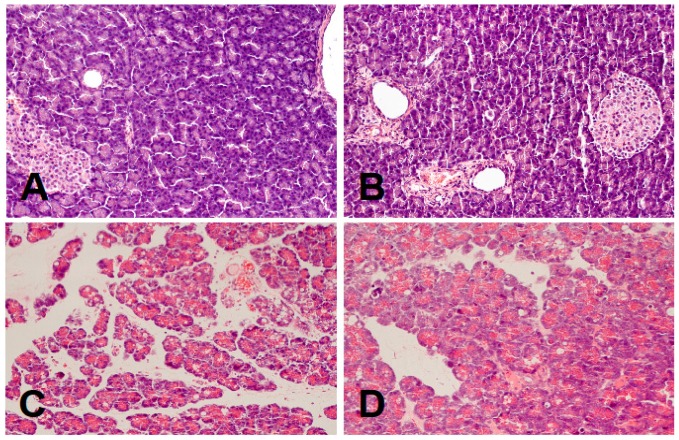
Representative morphological images of the pancreas observed in control saline treated rats (**A**); rats pretreated with acenocoumarol given the dose of 50 μg/kg/day without induction of acute pancreatitis (**B**); rats with cerulein-induced acute pancreatitis (**C**); and rats pretreated with acenocoumarol (given the dose of 50 μg/kg/day) before induction of acute pancreatitis by cerulein (**D**). Hematoxylin–eosin counterstain, original magnification 200×.

**Figure 4:**
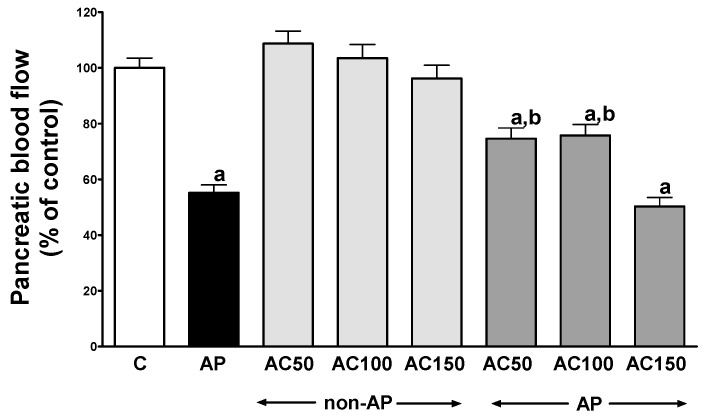
Impact of pretreatment with acenocoumarol on pancreatic blood flow in rats with or without cerulein-induced pancreatitis. Key: C = control; AP = cerulein-induced acute pancreatitis; non-AP = groups without induction of acute pancreatitis; AC = acenocoumarol; 50 = 50 μg/kg/day; 100 = 100 μg/kg/day; 150 = 150 μg/kg/day. Mean ± SEM. *n* = 10 in each group of rats. ^a^ *p* < 0.05 compared to control; ^b^ *p* < 0.05 compared to AP alone.

**Figure 5:**
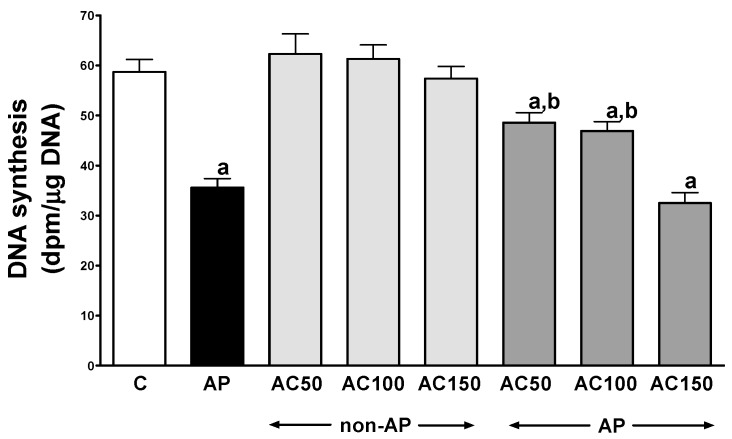
Impact of pretreatment with acenocoumarol on pancreatic DNA synthesis in rats with or without cerulein-induced pancreatitis. Key: C = control; AP = cerulein-induced acute pancreatitis; non-AP = groups without induction of acute pancreatitis; AC = acenocoumarol; 50 = 50 μg/kg/day; 100 = 100 μg/kg/day; 150 = 150 μg/kg/day. Mean ± SEM. *n* = 10 in each group of rats. ^a^ *p* < 0.05 compared to control; ^b^ *p* < 0.05 compared to AP alone.

**Figure 6:**
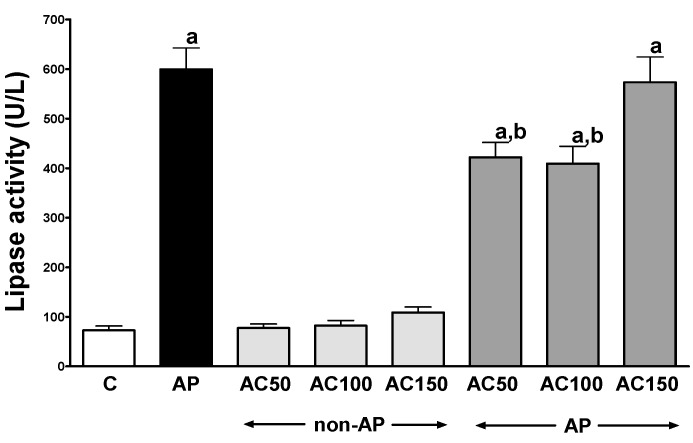
Impact of pretreatment with acenocoumarol on serum activity of lipase in rats with or without cerulein-induced pancreatitis. Key: C = control; AP = cerulein-induced acute pancreatitis; non-AP = groups without induction of acute pancreatitis; AC = acenocoumarol; 50 = 50 μg/kg/day; 100 = 100 μg/kg/day; 150 = 150 μg/kg/day. Mean ± SEM. *n* = 10 in each group of rats. ^a^ *p* < 0.05 compared to control; ^b^ *p* < 0.05 compared to AP alone.

**Figure 7:**
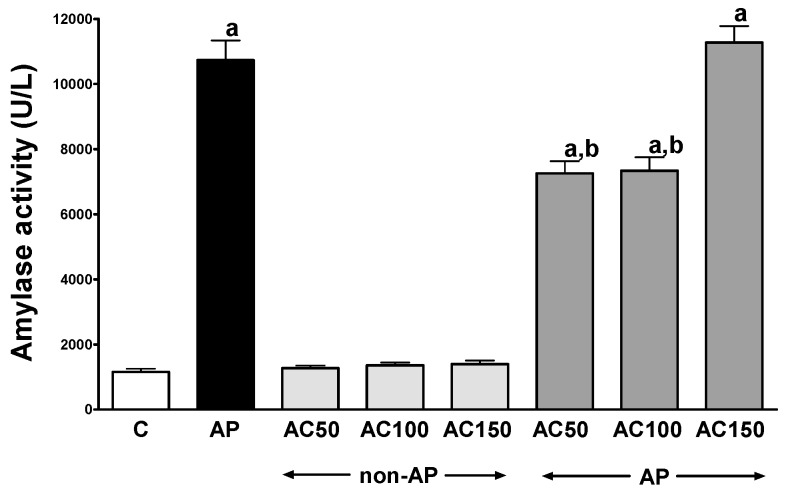
Impact of pretreatment with acenocoumarol on serum activity of amylase in rats with or without cerulein-induced pancreatitis. Key: C = control; AP = cerulein-induced acute pancreatitis; non-AP = groups without induction of acute pancreatitis; AC = acenocoumarol; 50 = 50 μg/kg/day; 100 = 100 μg/kg/day; 150 = 150 μg/kg/day. Mean ± SEM. *n* = 10 in each group of rats. ^a^ *p* < 0.05 compared to control; ^b^ *p* < 0.05 compared to AP alone.

**Figure 8:**
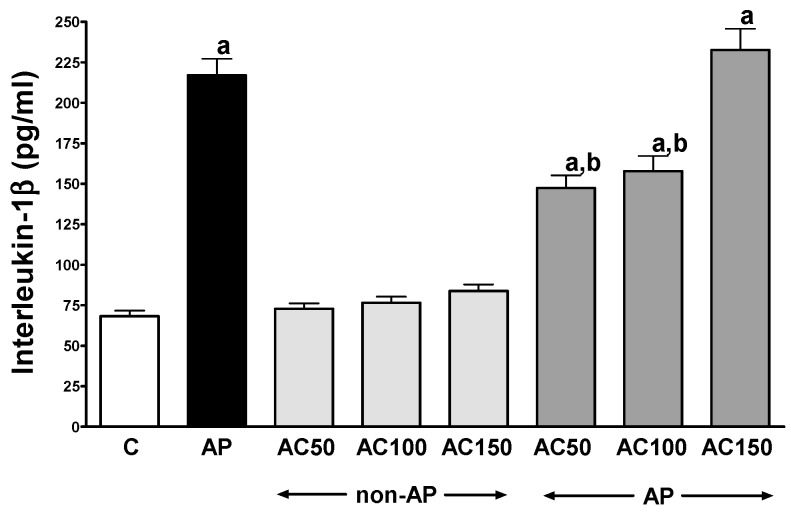
Impact of pretreatment with acenocoumarol on serum concentration of interleukin-1β in rats with or without cerulein-induced pancreatitis. Key: C = control; AP = cerulein-induced acute pancreatitis; non-AP = groups without induction of acute pancreatitis; AC = acenocoumarol; 50 = 50 μg/kg/day; 100 = 100 μg/kg/day; 150 = 150 μg/kg/day. Mean ± SEM. *n* = 10 in each group of rats. ^a^ *p* < 0.05 compared to control; ^b^ *p* < 0.05 compared to AP alone.

**Figure 9:**
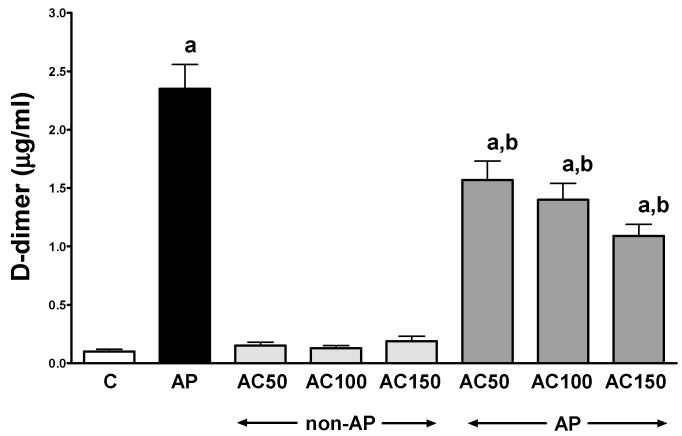
Impact of pretreatment with acenocoumarol on plasma D-Dimer concentration in rats with or without cerulein-induced pancreatitis. Key: C = control; AP = cerulein-induced acute pancreatitis; non-AP = groups without induction of acute pancreatitis; AC = acenocoumarol; 50 = 50 μg/kg/day; 100 = 100 μg/kg/day; 150 = 150 μg/kg/day. Mean ± SEM. *n* = 10 in each group of rats. ^a^ *p* < 0.05 compared to control; ^b^ *p* < 0.05 compared to AP alone.

In control saline-treated rats without induction of AP, international normalized ratio (INR) reached a value of 1.08 ± 0.10 (Figure 1) while the average weight of the pancreas was 618 ± 22 g (Figure 2). Morphological features showed that the pancreas in this group of animals exhibits regular histology without damage of the gland (Figure 3 and Table 1). In rats without induction of AP, administration of acenocoumarol given for seven days caused a dose-dependent increase in INR (Figure 1). Acenocoumarol given at the dose of 50, 100 or 150 μg/kg/dose caused around three-, four- and six-fold increase in INR, respectively. In these rats, acenocoumarol given at the doses used did not cause a statistically significant effect on the weight of the pancreas (Figure 2), pancreatic blood flow (Figure 4) and pancreatic DNA synthesis (Figure 5). In addition, serum activity of lipase (Figure 6) and amylase (Figure 7), serum concentrations of interleukin-1β (IL-1β) (Figure 8), and plasma D-dimer concentration (Figure 9) were not affected by acenocoumarol given in rats without causing pancreatitis. Morphological features showed that acenocoumarol given alone caused in half of cases slight interlobular edema of the pancreas (Table 1). Moreover, in contrast to lower doses, acenocoumarol given at the dose of 150 µg/kg/dose led to appear single hemorrhagic foci in pancreatic tissue in half of animals without induction of pancreatitis (Table 1).

Intraperitoneal administration of cerulein led to the development of edematous AP in all tested rats (Table 1). Morphological features showed interlobular a moderate or severe intralobular edema. This change was accompanied by a scare or moderate perivascular and scare diffuse inflammatory leukocyte infiltration. Vacuolization was observed in more than 25% of acinar cells. Necrosis and hemorrhage were not observed (Figure 3 and Table 1). Histological signs of pancreatic damage were associated with functional and biochemical changes typically present in cerulein-induced AP. The weight of the pancreas increased by 76% (Figure 2), while the pancreatic blood flow (Figure 4) and pancreatic DNA synthesis (Figure 5) decreased by 45% and 40%, respectively. Intraperitoneal administration of cerulein also resulted in more than an eight-fold increase in serum activity of lipase (Figure 6) and more than a nine-fold increase in serum activity of amylase (Figure 7). Serum concentration of pro-inflammatory IL-1β reached around 320% of a value observed in control rats (Figure 8), whereas INR (Figure 1) and plasma D-Dimer concentration (Figure 9) increased by 48% and 2350%, respectively.

Pretreatment with acenocoumarol given at the dose of 50 or 100 μg/kg/dose attenuated the development of acute cerulein-induced pancreatitis. Histological examination showed a reduction in pancreatic edema, inflammatory infiltration and vacuolization of acinar cells (Table 1 and Figure 3). In addition, acenocoumarol given at the dose of 50 or 100 μg/kg/dose significantly decreased the pancreatitis-evoked increase in pancreatic weight (Figure 2) serum activity of lipase (Figure 6) and amylase (Figure 7), and serum concentration of pro-inflammatory IL-1β (Figure 9). These effects were accompanied by a partial but statistically significant reversal of cerulein-induced decline in pancreatic blood flow (Figure 4) and pancreatic DNA synthesis (Figure 5).

In contrast to effects of low doses of acenocoumarol, acenocoumarol given at the dose of 150 μg/kg/dose was without beneficial effect on the pancreatitis-evoked changes of pancreatic weight (Figure 2), pancreatic blood flow (Figure 4), pancreatic DNA synthesis (Figure 5) serum activity of pancreatic enzymes (Figure 6 and Figure 7) and serum concentration of IL-1β (Figure 8). Morphological features showed that pretreatment with acenocoumarol given at the dose of 150 μg/kg/dose was without beneficial effect on the pancreatitis-evoked pancreatic inflammatory infiltration (Table 1). Moreover, pretreatment with acenocoumarol given at the dose of 150 μg/kg/dose increased the pancreatitis-evoked pancreatic edema and number of hemorrhages. Only the number of acinar cells with signs of vacuolization was partially reduced (Table 1).

As in animals without pancreatitis, pretreatment with acenocoumarol resulted in dose dependent increase in INR (Figure 1). Acenocoumaral given at all doses used caused a similar and statistically significant reduction in the pancreatitis-evoked increase in plasma D-Dimer concentration (Figure 9).

## 3. Discussion

In our present study, we investigated the impact of pretreatment with acenocoumarol on the development of cerulein-induced AP in rats. This experimental model of AP leads to the development of mild edematous pancreatitis evoked by primary non-vascular mechanism [23]. To our knowledge, our investigation is the first report showing that low doses of acenocoumarol exhibit a protective effect on the pancreas in AP evoked by cerulein. Importantly, our observation has been supported by histological, functional and biochemical evidence.

The beneficial effect of pretreatment with low doses of acenocoumarol in rats with AP was manifested by a reduction in morphological signs of pancreatic damage including pancreatic edema, vacuolization of acinar cells and inflammatory leukocyte infiltration of pancreatic tissue. A reduction in pancreatic edema was also found as a decrease in the pancreatitis-evoked increase in pancreatic weight. Previous studies have shown that the onset of AP is associated with accumulation of leukocytes within the pancreas and initiation of the local inflammatory response. Activation of neutrophils leads to their adhesion to endothelial cells in pancreatic microcirculation causing the reduction in pancreatic blood flow, inflammatory infiltration of pancreatic tissue and production of pro-inflammatory cytokines within this organ [24,25,26]. In severe cases of AP, it leads to the development of necrotizing AP, systemic inflammatory response syndrome (SIRS) and multiple organ failure (MOF) [27].

In our present study, acenocoumarol-related reduction in the inflammatory leukocyte infiltration of pancreatic tissue in rats with AP was in harmony with a decrease in serum concentration of interleukin-1β (IL-β). Activation of leukocytes leads to the release of pro-inflammatory cytokines and is responsible for the development of local and systemic inflammatory response in AP, as well as may lead to chronic inflammation and promotion of pancreatic fibrosis [28,29]. The level of pro-inflammatory cytokines affects the severity of AP [28]. IL-1β plays a key role in the induction of local inflammatory response and systemic acute phase response [30]. This cytokine stimulates the release of next members of pro-inflammatory cascade, such as tumor necrosis factor-α (TNF-α), platelet-activating factor, prostaglandins and other pro-inflammatory interleukins [28,29,30]. The important role of leukocyte activation and IL-1β release in development and course of AP has been additionally evidenced by observation that administration of IL-1 receptor antagonist prevents a serum rise in IL-6 and TNF-α, and decreases the severity of AP [31]. In our present study, similarly to other previous reports [28,32,33], we have detected the increase in serum levels of IL-1β in rats with AP. We have also found that pretreatment with low doses of acenocoumarol resulted in a significant reduction of serum IL-1β concentration and limitation of AP severity. These important findings are consistent with an earlier report on the acenocoumarol-related reduction in IL-1β release in ischemia/reperfusion-induced AP and indicate that acenocoumarol is a universal protective agent in AP [34].

The increase in serum activity of pancreatic digestive enzymes, lipase and amylase is a well-known index of AP severity with high sensitivity and specificity [35,36]. In our present study, rats with cerulein-induced pancreatitis demonstrated an eight- and nine-fold increase in serum activity of lipase and amylase, respectively, compared to the control. Pretreatment with acenocoumarol given at the dose of 50 or 100 μg/kg/day reduced the pancreatitis-evoked increase in serum activity of lipase and amylase. This effect seems to be a result and, at least in part, a mechanism of protective properties of acenocoumarol in the pancreas. Study performed by Keck et al. [37] showed that presence of active pancreatic digestive enzymes in the circulation up-regulates the expression of adhesion molecules on leukocytes and endothelial cells, leading to increase in leukocyte-endothelial interaction and disturbance pancreatic microcirculation.

In harmony with our histological and biochemical observations supporting the protective impact of acenocoumarol on the pancreas in cerulein-induced acute AP is its effect on pancreatic DNA synthesis. DNA synthesis is an index of cell vitality and proliferation. Reduction in pancreatic DNA synthesis is well-correlated with pancreatic damage in acute pancreatitis [38,39,40]. In our present study, acenocoumarol given alone without induction of AP was without effect on pancreatic DNA synthesis. This observation indicates that acenocoumarol given alone in doses used does not affect pancreatic cell vitality and proliferation. Induction of AP by cerulein administration led to reduction of pancreatic DNA synthesis by around 45% and this effect was reversed by pretreatment with low doses of acenocoumarol. This our result is similar to that observed in rats pretreated with acenocoumarol before induction of AP evoked by pancreatic ischemia followed by reperfusion [34] showing that pancreatoprotective effect of acenocoumarol is independent of primary cause of AP.

Disorders in pancreatic microcirculation are observed in all cases of AP, independently to a primary etiology of this disease. Subsequently, it leads to activation of leukocytes and clotting in pancreatic blood vessels, as well as to liberation of pro-inflammatory cytokines and pancreatic digestive enzymes [26,41,42,43,44]. Microvascular impairment in the course of AP may be limited to pancreatic circulation, but very often it occurs within the microcirculation of other organs such as the kidney, lung, colon or liver [44]. On the other hand, the improvement of pancreatic blood flow inhibits the development of acute pancreatitis and accelerates the recovery in this disease [45,46,47]. In the present study we have confirmed that cerulein-induced AP decreases pancreatic blood flow. Our study has shown that pretreatment with acenocoumarol, given at the low dose of 50 or 100 μg/kg/day, improves pancreatic blood flow in rats exposed to cerulein and this effect has been associated with reduction of severity of pancreatic damage. This observation indicates that protective effect of acenocoumarol in cerulein-induced acute pancreatitis involves improvement of pancreatic microcirculation. However, this effect seems to be secondary and related to anticoagulant activity of acenocoumarol.

Vitamin K is necessary for the correct synthesis of prothrombin and three other plasma-clotting factors, factor VII, IX and X. Normally, precursors of these clotting factors undergoes posttranslational carboxylation by γ-glutamyl-carboxylase in liver microsomes prior to secretion into plasma. The activity of y-glutamyl carboxylase requires the presence of vitamin K hydroquinone, which is oxidized to vitamin K epoxide during carboxylation of precursors of coagulation factors. Vitamin K epoxide is than reconverted to a reduced form of vitamin K by vitamin K epoxide reductase. Acenocoumarol, as other vitamin K antagonists act as a competitive inhibitor of vitamin K reductase, leading to reduction in a plasma level of functional precursors of vitamin K-dependent clotting factors and the appearance, in the circulation, of biologically inactive precursors known as protein induced by vitamin K absence/antagonist-II (PIVKA-II) or des-γ carboxyprothrombin (DCP) [48,49].

Activation of coagulation has been shown to stimulate the inflammatory responses by the presence of active clotting factors (especially thrombin, factor Xa and tissue factor-factor VIIa complex), mediators released from platelets and promotion of cell-cell interaction [1,2,3]. Acenocoumarol, as other vitamin K antagonists, decreases the liver production of prothrombin and reduces formation of thrombin after induction of coagulation. Thrombin promotes blood coagulation, but it also serves as a signaling molecule by binding to protease-activated receptors (PARs) [50,51]. The presence of PARs has been found inter alia on platelets, endothelial cells, and also various immune cells such as lymphocytes, macrophages, monocytes, dendritic cells and mast cells [50,51,52]. In platelets, pro-inflammatory effect of thrombin is related to alteration of platelets shape to active phenotype and release of platelet factors. Furthermore, thrombin liberates the fibrinogen receptor GPIIb-IIIa integrin complex and P-selectin, as well as mobilizes the CD40 ligand to the platelet surface. Moreover, CD40 ligand induces endothelial cells to secrete chemokines and to express adhesion molecules, leading to generation of signals for recruitment and extravasation of leukocytes [51]. Acting directly on PARs on endothelial cells, thrombin and other proteases of the coagulation-fibrinolysis system change shape of these cells into a pro-inflammatory phenotype, increase vascular permeability, mobilize adhesive molecules and stimulate the production of cytokines leading to the local accumulation of platelets and leukocytes [52]. In addition, fibrinolytic proteases exhibit pro-inflammatory effects. Plasmin forms fibrin degradation products, which acting on toll-like receptor-4 (TLR-4) can release latent matrix-bound growth factors. Furthermore, proteases that convert plasminogen into plasmin, such as urokinase plasminogen activator (uPA), demonstrate the plasmin-independent pro-inflammatory action by binding to their receptors and co-receptors [5].

Induction of AP by cerulein led to increase in INR and plasma D-Dimer concentration. This observation indicates that development of edematous cerulein-induced AP is associated with activation of coagulation and formation of thrombi within the circulation, and this process is followed by fibrinolysis. This finding is in harmony with previous animal and clinical studies showing that AP activates coagulation and may lead to the development of consumptive coagulopathy [6,53,54]. D-Dimer is a product of plasmin-induced degradation of stabilized fibrin [55,56] and for this reason it is recognized as a marker of fibrinolysis activation [7,54,57].

Our study has shown that pretreatment with acenocoumarol dose-dependently increases INR and this effect reached a similar rate in animals with or without subsequent induction of AP. On the other hand, pretreatment with acenocoumarol significantly reduced the pancreatitis-evoked increase in plasma D-Dimer concentration. These findings indicate that pretreatment with acenocoumarol reduces the level of clotting factors in plasma and decreases the activation of coagulation during induction of AP. It inhibits formation of thrombin and reduces creation of D-Dimer, a product of fibrinolysis.

In contrast to protective effects of acenocoumarol given at the dose of 50 or 100 μg/kg/day, our present study has shown that administration of this vitamin K antagonist at the dose of 150 μg/kg/day doses not exhibit any protective effect against the development of cerulein-induced AP. This finding is most likely a result of excessive reduction in blood coagulation leading to excavation of blood from blood vessels and disturbance of general and organ circulation. This concept is supported by our present observation that pretreatment with acenocoumarol given at the dose of 150 μg/kg/day causes a five-fold increase in INR. Lack of protective effect of pretreatment with acenocoumarol given at the dose of 150 μg/kg/day is also in harmony with previous observation that this dose of acenocoumarol does not prevent the development of ischemia/reperfusion-induced AP [34].

From the clinical point of view, the protective effect of acenocoumarol in acute pancreatitis could be useful in preventing the development of acute pancreatitis following endoscopic retrograde cholangiopancreatography (ERCP). ERCP, as an invasive procedure, carries a significant risk to the patients. Post-ERCP complication rate vary widely depending on the complexity of the procedure and the predisposition of the patient. Acute pancreatitis is the most frequent complication of ERCP, which is reported to occur in 2%–10% of unselected patients and up to 8%–40% in high-risk patients [58,59,60]. Apart from acute pancreatitis, other relatively frequent post-ERCP complications are hemorrhage, cholangitis and perforation [58,60]. Post-ERCP bleeding has been reported in up to 4.5% of patients [58,60]. Several studies have suggested that defined risk factors for bleeding are coagulopathy, anticoagulation within three days of sphincterotomy, cholangitis before ERCP and bleeding during initial endoscopic sphincterotomy [58]. Our current study has shown that acenocoumarol given at the dose 50 µg/kg/dose increases INR to 3. This value is associated with some risk of bleeding. Those findings may suggest that the possibility of the use of acenocoumarol in the prevention of ERCP-induced pancreatitis is questionable. On the other hand, there is the study showing that in multivariate analysis anticoagulants are not a statistically significant independent risk factor for post-ERCP bleeding [60]. These data indicate that the potential utility of acenocoumarol in the prevention of post-ERCP pancreatitis is ambiguous and needs further research in this area.

## 4. Materials and Methods

### 4.1. Animals and Treatment

All studies followed an experimental protocol approved by the Committee for Research and Animal Ethics of the Jagiellonian University and the First Local Commission of Ethics for the Care and Use of Laboratory Animals in Cracow (Permit Number 4/2013 released on 16 January 2013).

Studies were carried out on 80 male Wistar rats weighing 160–180 g, which were housed in cages with wire-mesh bottoms in a windowless colony room. Temperature was adjusted at 22 ± 1 °C with relative humidity of 50% ± 10%, and 12 h:12 h light:dark photoperiod. During the study animals had free access to food and water.

Following a one-week period of acclimation to their new environment, rats were randomly divided into eight equal experimental groups: (1) saline-treated control rats; (2) rats with cerulein-induced AP; (3–5) rats without induction of AP pretreated with acenocoumarol given at the dose of 50, 100 or 150 μg/kg/dose; and (6–8) rats pretreated with acenocoumarol given at the dose of 50, 100 or 150 μg/kg/dose before induction of AP by cerulein administration.

AP was induced by cerulein (Sigma-Aldrich, GmbH, Steinheim, Germany) given intraperitoneally (i.p.) 5 times with 1 h intervals at a dose of 50 μg/kg per injection (group 2, 6, 7 and 8). At the same time, animals from groups without induction of AP were treated i.p. with saline (group 1, 3, 4 and 5).

Acenocoumarol (Acenocumarol WZF, Warszawskie Zakłady Farmaceutyczne Polfa S.A., Warsaw, Poland) at the dose of 50, 100 or 150 μg/kg/dose was administered intragastrically (i.g.) once a day for 7 day before induction of AP (group 6, 7 and 8) or before intraperitoneal administration of saline (rats without induction of AP: group 3, 4 and 5). Animals without administration of acenocoumarol were treated i.g. for 7 days with saline. Acenocoumarol was given at the dose of 50, 100 or 150 μg/kg/dose because previous studies [34] showed that these doses caused an increase of international normalized ratio (INR) to a range between 2.5 and 3.5. This value of INR is recommended in the most clinical conditions related to coagulation disorders [61].

### 4.2. Determination of Pancreatic Blood Flow

Immediately after the last i.p. injection of cerulein or saline, rats were anesthetized with ketamine (50 mg/kg i.p., Bioketan, Vetoquinol Biowet, Gorzów Wielkopolski, Poland) and experiment was terminated. After opening the abdominal cavity the pancreas was exposed and blood flow in this organ was determined using a laser Doppler flowmeter (PeriFlux 4001 Master Monitor, Perimed AB, Järfälla, Sweden) in accordance with the method described previously in detail [62,63]. Data were presented as percent change from value obtained in control saline-treated rats without induction of AP.

### 4.3. Biochemical Analysis

After the measurement of pancreatic blood flow, blood samples were taken from the abdominal aorta. The prothrombin time measured as international normalized ratio (INR) was determined in fresh blood, using Alere INRatio^®^ 2 PT/INR Monitoring Systems and Alere INRatio^®^ PT/INR Monitoring System Test Strips (Alere San Diego, Inc., San Diego, CA, USA).

Plasma D-Dimer concentration was determined using an immunoturbidimetric assay (Innovance D-Dimer Assay, Simens Healthcare GmbH, Marburg, Germany) on automatic coagulation analyzer BCS XP System (Simens Healthcare Diagnostics, Erlangen, Germany).

Serum lipase and amylase activity was determined with a Kodak Ectachem DT II System analyzer (Eastman Kodak Company, Rochester, NY, USA) using Lipa and Amyl DT Slides (Vitros DT Chemistry System, Johnson & Johnson Clinical Diagnostic, Inc., Rochester, NY, USA).

Serum concentration of interleukin-1β (IL-1β) was measured using the Rat IL-1β Platinum Elisa (Bender MedSystem GmbH, Vienna, Austria).

### 4.4. Determination of Pancreatic DNA Synthesis

After blood collection, the pancreas was cut out from its atachment to other organs and weighed. Samples of pancreatic tissue were collected for determination of pancreatic DNA synthesis and histological examination. The rate of pancreatic DNA synthesis was measured by assessing the incorporation of labeled thymidine ((6-^3^H)-thymidine, 20–30 Ci/mmol, Institute for Research, Production and Application of Radioisotopes, Prague, Czech Republic) into DNA, as described previously in detail [64,65]. Rate of DNA synthesis was expressed as disintegrations of labeled thymidine per minute per microgram DNA (dpm/μg DNA).

### 4.5. Histological Examination of Pancreatic Damage

Microscopic examination of pancreatic tissue damage was performed in hematoxylin and eosin (H&E) stained slides by two experienced pathologists as described previously in detail [66]. Histological grading of pancreatic edema, leukocyte inflammatory infiltration, vacuolization of acinar cells, hemorrhages, and pancreatic necrosis was made using a scale ranging from 0 to 3. Results of microscopic examination of the pancreas were expressed as the most frequent histological score (mode) in each experimental group.

### 4.6. Statistical Analysis

Statistical analysis was made by analysis of variance followed by Tukey’s multiple comparison test using GraphPadPrism (GraphPad Software, San Diego, CA, USA). The results were presented as means ± SEM. Each experimental group consisted of ten animals. A difference with a *p* value of less than 0.05 was considered significant.

## 5. Conclusions

In conclusion, we can say that results of our present experiments have indicated that low doses of acenocoumarol exerts a pronounced protective effect on the pancreas and inhibits the development of cerulein-induced AP. These findings taken together with a previous report showing preventive effect of low doses of acenocoumarol in ischemia/reperfusion-induced AP indicate that protective effect of low doses of acenocoumarol in the pancreas is universal and independent of the primary cause of AP.

## Figures and Tables

**Table 1:** Impact of pretreatment with acenocoumarol on histological signs of pancreatic damage in rats with or without cerulein-induced pancreatitis.

Groups	Edema (0–3)	Inflammatory Infiltration (0–3)	Vacuolization (0–3)	Necrosis (0–3)	Hemorrhages (0–3)
Control	0	0	0	0	0
AP	2–3	1-2	3	0	0
AC 50	0–1	0	0	0	0
AC 100	0–1	0	0	0	0
AC 150	0–1	0	0	0	0-1
AC 50 + AP	1–2	1	2	0	0
AC 100 + AP	1–2	1	2	0	0
AC 150 + AP	3	1–2	2–3	0	1

Numbers represent the predominant histological grading in each experimental group. Key: Control = rats without induction of AP or treatment with acenocoumarol; AP = cerulein-induced acute pancreatitis; AC = acenocoumarol; 50 = 50 μg/kg/day; 100 = 100 μg/kg/day; 150 = 150 μg/kg/day.
