# Correction: Warzecha, Z. et al. Protective Effect of Pretreatment with Acenocoumarol in Cerulein-Induced Acute Pancreatitis. *Int. J. Mol. Sci.* 2016, *17*, 1709

**DOI:** 10.3390/ijms20122893

**Published:** 2019-06-13

**Authors:** Zygmunt Warzecha, Paweł Sendur, Piotr Ceranowicz, Marcin Dembiński, Jakub Cieszkowski, Beata Kuśnierz-Cabala, Rafał Olszanecki, Romana Tomaszewska, Tadeusz Ambroży, Artur Dembiński

**Affiliations:** 1 Department of Physiology, Faculty of Medicine, Jagiellonian University Medical College, 16 Grzegórzecka St., 31-531 Cracow, Poland; p.send@interia.pl (P.S.); mpcerano@cyf-kr.edu.pl (P.C.); jakub.cieszkowski@uj.edu.pl (J.C.); mpdembin@cyf-kr.edu.pl (A.D.); 2 Department of Anesthesiology and Intensive Therapy, Faculty of Medicine, Jagiellonian University Medical College, 31-501 Cracow, Poland; 3 The Second Department of General Surgery, Faculty of Medicine, Jagiellonian University Medical College, 31-501 Cracow, Poland; mpmdembi@cyf-kr.edu.pl; 4 Department of Clinical Biochemistry, Faculty of Medicine, Jagiellonian University Medical College, 31-501 Cracow, Poland; mbkusnie@cyf-kr.edu.pl; 5 Department of Pharmacology, Faculty of Medicine, Jagiellonian University Medical College, 31-531 Cracow, Poland; rafal.olszanecki@uj.edu.pl; 6 Department of Pathology, Faculty of Medicine, Jagiellonian University Medical College, 31-531 Cracow, Poland; romatom@mp.pl; 7 Faculty of Physical Education and Sport, University of Physical Education, 31-571 Cracow, Poland; tadek@ambrozy.pl

We would like to submit the correction to our published paper [1]. The reason for the correction is an error in the histological image presented in the published article. Two histological images (Figure 3A,B in [1]) are incorrect and, therefore, they need to be replaced with the correct new figure (Figure 1). 

The reported error does not have any material impact on the final results and conclusions of our published paper. We apologize for this inconvenient situation. 

## Figures and Tables

**Figure 1:**
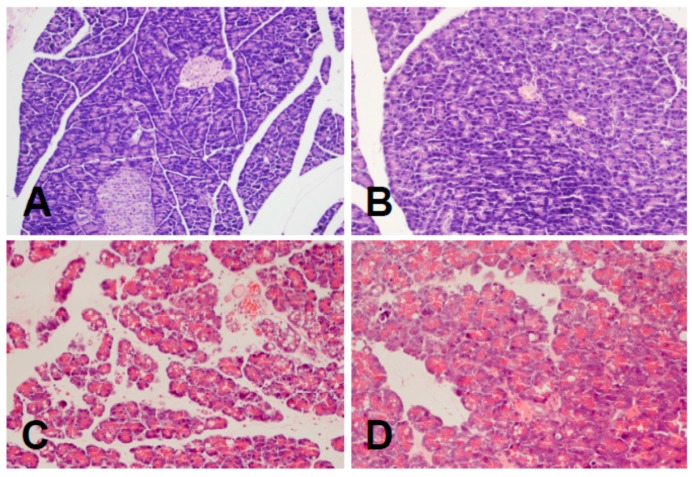
Representative morphological images of the pancreas observed in control rats treated with saline (**A**); rats pretreated with acenocoumarol at the dose of 50 μg/kg/day without induction of acute pancreatitis (**B**); rats with cerulein-induced acute pancreatitis (**C**); rats pretreated with acenocoumarol (given the dose of 50 μg/kg/day) before induction of acute pancreatitis by cerulein (**D**). Hematoxylin-eosin counterstain, original magnification 200×.
